# Can Endothelial Glycocalyx Be a Major Morphological Substrate in Pre-Eclampsia?

**DOI:** 10.3390/ijms21093048

**Published:** 2020-04-26

**Authors:** Marina M. Ziganshina, Ekaterina L. Yarotskaya, Nicolai V. Bovin, Stanislav V. Pavlovich, Gennady T. Sukhikh

**Affiliations:** 1 National Medical Research Center for Obstetrics, Gynecology and Perinatology named after Academician V.I. Kulakov of the Ministry of Health of Russian Federation, 117997 Moscow, Russia; 2 M.M. Shemyakin and Y.A. Ovchinnikov Institute of Bioorganic Chemistry, Russian Academy of Sciences, 117997 Moscow, Russia; 3 Federal State Autonomous Educational Institution of Higher Education I.M. Sechenov First Moscow State Medical University under the Ministry of Health of the Russian Federation (Sechenov University), 119991 Moscow, Russia

**Keywords:** pregnancy, pre-eclampsia, endothelium, endothelial glycocalyx, systemic inflammatory response

## Abstract

Today pre-eclampsia (PE) is considered as a disease of various theories; still all of them agree that endothelial dysfunction is the leading pathogenic factor. Endothelial dysfunction is a sequence of permanent immune activation, resulting in the change of both the phenotype and the functions of an endothelial cell and of the extracellular layer associated with the cell membrane—endothelial glycocalyx (eGC). Numerous studies demonstrate that eGC mediates and regulates the key functions of endothelial cells including regulation of vascular tone and thromboresistance; and these functions are disrupted during PE. Taking into account that eGC and its components undergo alterations under pathological conditions leading to endothelial activation, it is supposed that eGC plays a certain role in pathogenesis of PE. Envisaging the eGC damage as a key factor of PE, might be a new approach to prevention, treatment, and rehabilitation of patients with PE. This approach could include the development of drugs protecting eGC and promoting regeneration of this structure. Since the issue of PE is far from being solved, any effort in this direction might be valuable.

## 1. Background

Pre-eclampsia (PE) is one of the most serious complications in pregnancy, which takes the third place in the list of causes of maternal mortality, and is the major cause of the neonatal morbidity and mortality [1]. PE is currently regarded as multisystemic pathological condition with clinicalmanifestations starting after the 20th week of pregnancy. It is characterized by arterial hypertension in combination with proteinuria, and often by edema and signs of polyorganic/polysystemic insufficiency [2,3]. Placental ischemia acts as an initiating factor in PE pathogenesis and may be caused by inadequate gestational remodeling of maternal spiral arteries, due to compromised processes of trophoblast invasion, as well as by the blockade of spiral arteries due to congenital or acquired thrombophilia [4].

Development of placental ischemia is characterized by an increase of apoptosis in the placental structures and entry of necrotic debris and microparticles of trophoblastic origin into the maternal blood. These changes initiate the launch of systemic inflammatory response (SIR): activation of immune cells and the complement system, synthesis of proinflammatory cytokines, and, consequently, development of endothelial dysfunction [5,6,7].

Endothelial dysfunction is thought to play a major role in pathogenesis of PE, since endothelium is a unique monolayer of the cells located in the vessels of the main target organs (liver, kidney, uterus, placenta, central nervous system (CNS)); pathologic changes in these organs determine the maternal and fetal outcomes [8]. Endothelial dysfunction is a consequence of chronic activation, resulting in changes not only in the phenotype of endothelial cells, but also in impairment of their control over vascular tone and permeability, as well as over intravascular hemostasis [9,10].

Pro-inflammatory impact on endothelium will inevitably affect the molecular structure of endothelial glycocalyx (eGC) [11], which is a highly organized polyanionic complex formed by lipid-carbohydrate (membrane-anchored glycosphyngolipids), protein-carbohydrate (glycoproteins and proteoglycans) conjugates, and glycosaminoglycans (GAGs), located on the luminal surface of endothelial cells. The eGC proteoglycans are mostly syndecans, glypicans, and endocans. The carbohydrate part of these proteoglycans consists of glycosaminoglycan chains: heparan sulphates, chondroitin sulphates, dermatan sulphates. Heparan sulfates account for 50%–90% of all proteoglycans within the eGC. The binding and stabilizing structural unit of eGC is hyaluronic acid (HA) [12]. Compared to other cells, the glycocalyx of endothelial cells is much thicker, ranging from 0.2–0.5 μm in capillaries to 2–3 μm in small arteries and 4.5 μm in carotid arteries—this is much more than the length of the various cell adhesion molecules in their extended conformation. The high density of negative charges (due to the presence of carboxyl groups and sulfate residues in GAGs) gives the glycocalyx (GC) the properties of a loose gel that can slow down and regulate the passage of molecules and microvesicles from the blood stream to the cells. Moreover, this gel can act as a molecular sieve (similar to chromatographic media for gel permeation chromatography), allowing the passage of microvesicles and large macromolecules, but retaining small molecules in its pores [13]. Even more similarity to gel permeation chromatography arises from the fact that the liquid medium is constantly moving along the endothelial cells. Obviously, due to such physical properties, eGC is not a barrier, but rather a smooth operator of molecules and microvesicles transfers to and from endothelium; the abnormalities in the biosynthesis of eGC molecules can dramatically change its dispatching ability. Specific processes mediated by glycocalyx proteins and glycans play even more important role in eGC functioning. In particular, eGC retains galectins [14] which perform not only the role of adhesion molecules, but receptor functions as well.

Recent studies indicate that homeostatic functions of endothelium are ensured by the intactness of the eGC; its composition and structure significantly differ under the physiological and pathophysiological conditions [15,16,17]. The changes also affect the functions of eGC, which are equal to the functions of endothelial cells. Table 1 illustrates the functions of undamaged (intact) eGC under normal conditions, and those of the damaged (degraded) eGC, being shed and destructed under SIR, which to a certain extent accompanies any pathological process.

Abnormal changes of eGC can be linked to main clinical symptoms of PE: progressive endotheliosis, hypertension, tissue edema, disseminated intravascular coagulation (DIC) syndrome, impaired permeability of glomerular and hematoencephalic barriers (see Table 1). However, despite obvious parallels between the PE signs and the degradation of eGC, experimental and clinical studies of the relationship between these are very limited. This also applies to cardiovascular and inflammatory diseases, and kidney pathology. The main difficulties of such studies, remaining in spite of the advances in methodology [18], are the impossibility of direct in vivo visualization, and limitations of indirect in vitro evaluation of eGC [19].

**Table 1:** Regulatory role of endothelial glycocalyx in physiological and pathological conditions.

Function	Intact eGC	Damaged eGC
Regulation of mechanosensitivity of endothelial cells	Acts as a mechanotransducer, transmitting shear stress forces to endothelial cells. It accepts and dissipates the load caused by shear stress. The load is transferred to the side chains of proteoglycans, which transmit the torque to the core proteins and into the cell, activating the signal cascade reactions and the actin cytoskeleton. Stimulates the endothelial NO synthase, which regulates formation of endogenic nitric oxide synthase (eNOs)—the factor of vessels relaxation and cytoskeletal reorganization [20,21,22,23].	Decreases mechanosensitivity of endothelial cells. Main load of fluid shear stress affects the apical membrane of endothelial cells. Blocked shear-induced NO production, disruption of vascular tone regulation, deficient vasodilatation [15,23,24].
Regulation of vascular permeability	It has a structure of selective molecular sieve, with the filtration ability depending on the molecule size and charge. Facilitates permeability of low-molecular compositions. It is selectively permeable for macromolecules and performs barrier function [23,25,26].	Removal of key structural components of eGC leads to structural damage and increase of vessel permeability for high molecular plasma proteins (albumin) and development of tissue edema and loss of barrier function [27,28].
Regulation of interactions of blood cells with the vascular wall	Vascular protection via the inhibition of coagulation, leukocyte adhesion, and production and accumulation of active forms of oxygen. Outer layer of eGC bordering with blood: contains soluble components: growth factors, plasma proteins, including compounds, which support vascular thromboresistance (thrombomodulin, antithrombin III, endothelial protein C receptor, tissue factor pathway inhibitor) and compounds with antioxidant features (extracellular superoxide dismutase (ec-SOD)); shields cell adhesion molecules with branched chains of glycans; creates sufficient capillary resistance for circulating elements, thus excluding intercellular contacts [28,29,30].	Lacking or weak vascular protection. Shedding and destruction of the outer layer leads to: removal of thromboresistance factors and antioxidants; increased O_2_ production; denudation of cell adhesion molecules (CAMs) and exposure of glycans interacting with CAMs, which mediate rolling: emergence of hyperglycolyzed structures of glycans, neoantigens, which become targets for antibodies (AECA/APS) and immune cells [31,32,33,34,35].

Table 2 presents indirect studies of glycocalyx, including eGC in PE, providing evidence for some possible conclusions. First, the composition of GC in placental structures and the eGC capillaries were changed in PE; the most pronounced changes were noted in severe PE (Table 2, lines 3,9,12,13,15,16,19). Secondly, the contents of certain components of GC (HA, endocan, decorin, heparan sulfate) were increased in the blood of patients with PE; this may be a consequence of endothelial dysfunction, the shedding processes and destructive changes in eGC (Table 2, lines 1,7,10,11,13,14,18,19,21). Increased levels of free GAGs and proteoglycans in blood were also noted in patients with cardiovascular diseases, post cardiac arrest syndrome, sepsis, chronic kidney and venous disease [36,37,38,39,40,41]. In Hemolysis, Elevated Liver enzymes and Low Platelet count HELLP-syndrome, the increased content of eGC components in blood and the changes of glycocalyx in placental structures were also observed (Table 2, lines 4,5). However, the reported reduced level of syndecan 1 (sdc1) in blood of the PE patients contradicts this observation (Table 2, lines 6,16,17,21). This issue undoubtedly requires further investigation, since in patients with ischemic heart disease or heart failure, elevation of serum sdc1 has been associated with worsening cardiac and renal function; however, the causal relationship between degradation of eGC and clinical outcomes is unclear [38]. There is evidence of unchanged (Table 2, line 8, 9, 20) endocan contents, which may be due to specific characteristics of cohorts [42] and the paucity of the PE group [43]. Third, the urinary GAGs excretion and its increased urine contents (Table 2, line 2) were revealed in patients with PE and linked with decreased HSPGs (heparan sulfate proteoglycans) and CSPGs (chondroitin sulfate proteoglycans) contents in the glomerular basement membrane (GMB); this will disrupt the macromolecular organization of the GMB architecture with concomitant permeability changes and functional disorder of the GMB, thus leading to increased proteinuria [44,45].

Additionally, increased blood levels of HA and sdc1 were found in a case–control clinical trial in patients with chronic kidney diseases [37]. Damage to the eGC alters the permeability of multiple capillary beds: in the glomerulus this clinically shows as albuminuria. Generalized damage to eGC can therefore manifest as both albuminuria and increased systemic microvascular permeability. This triad including altered eGC, albuminuria, and increased systemic microvascular permeability occurs in a number of important diseases, such as diabetes, with accumulating evidence for a similar phenomenon in ischemia-reperfusion injury and infectious disease. In addition to indirect clinical evidence of impaired barrier function and eGC damage in PE, there is a number of experimental studies confirming the destruction of eGC and its components in rats with spontaneous albuminuric chronic kidney diseases [27], and in mice, receiving long-term hyaluronidase infusion [65].

If we assume that eGC is the main morphological substrate of PE, one may question, whether this glycopathology is primary (congenital) and manifests itself during pregnancy by PE development, or eGC damage is a result of ischemia in the placental tissue, leading, after a “point of no return”, to uncontrollable endothelial dysfunction. At present, there is no direct answer to these questions, since testing for glycopathology is not used in clinical practice. There are no studies comparing the glyco-gene panel in patients whose pregnancy completed successfully or was complicated by one of great obstetrical syndromes, e.g., PE. Anyway, the need of pathogenetic therapy or correction of the pathological condition caused by destabilization of eGC is obvious.

Most likely, early PE, which is associated with placentation disorder, would not benefit from therapy, aimed at the protection and regeneration of eGC, since organic dysmorphogenesis can hardly be overcome. Under permanent exposure to a pathogenetic factor, development of SIR and endothelial dysfunction would inevitably lead to premature birth. Late PE associated with the mother’s underlying condition is more likely to respond to eGC targeted therapy, because patients already receive specific treatment of the pre-existing disease. However, such therapy might be appropriate in both forms of PE, for restoration of endothelial function and prevention of the related long-term morbidity, since PE is known to be a risk factor for subsequent cardiovascular disease [66]. Ability of a number of drugs to stabilize the glycocalyx, enhance the endothelial surface layer, and to promote regeneration of eGC confirm the key role of eGC in pathogenesis of endothelial dysfunction in various diseases [16,38,67].

Currently, the list of drugs and biologically active molecules that were used in vitro in cell and animal models for protection and regeneration of eGC, is quite impressive. However, clinical studies of pathogenetic target therapy are lacking. There is evidence of a stimulating effect of a number of drugs on restoration of eGC after damage in various pathological conditions (Table 3). In particular, protective and regenerating effects of fresh frozen plasma (FFP) on eGC after massive bleeding in experimental animal models was noted. The review by Barelli S and Alberio L [68] demonstrate that FFP protects endothelium through restoration of the expression of sdc1, which is necessary for regeneration of eGC.

In mice with knockout of the Sdc-1^-/-^ gene, in contrast to wild-type mice, FFP did not show any protective effect. It is believed that the effects of FFP are caused by certain components: albumin and adiponectin. Removal of albumin from the bathing media in vitro induced collapse or shedding of glycocalyx. Probably, electrostatic interaction between arginine residues on albumin and negatively charged GAGs in the glycocalyx, can stabilize the glycocalyx structure. Albumin is one of the primary carriers of the phospholipid sphingosine-1-phosphate (S1P). The S1P-dependent protective effect of plasma on human umbilical vein endothelial cells (HUVECs) was demonstrated in the models of trauma and hemorrhagic shock [91]. It was alternatively suggested that S1P plays a critical role in protecting the glycocalyx via S1P1 and inhibits the protease activity-dependent shedding of CS, HS, and the syndecan-1 ectodomain [92]. However, it was noted that only early use of plasma in hemorrhagic shock may exert a clinically significant beneficial effect by preserving or even restoring the glycocalyx and, therefore, maintaining critical endothelial functions [68]. There is limited data on the effectiveness of FFP and its specific components in PE (Table 3, lines 1 and 2). Low level of adiponectin in the blood of patients with PE was found to be associated with endothelial dysfunction [93] and placental disorders; this may support further clinical use of adiponectin as a biomarker, therapeutic target, or therapeutic agent against the disease [94]. Similarly, S1P, according to several studies, is considered as a biomarker of cardiovascular disease, hypertension, and pregnancy-related complications like PE [95,96]. It was also reported that S1P inhibited the differentiation of primary human isolated cytotrophoblasts into syncytiotrophoblasts [97], and extravillous trophoblast migration [98]. Though there is no direct evidence of the impact of plasma and its components on eGC in PE, it can be presumed that the improvement of the disease course might result from the “treatment” of eGC by these agents.

A promising approach from the standpoint of etiotropic therapy aimed to protect eGC, is the use of anticoagulant drugs: heparin (unfractionated heparin (UFH), low-molecular-weight heparin (LMWH)) and heparinoids (sulodexide) [99]. Heparin is very similar to heparan sulfate, which is the main structural component of eGC. Heparinoids are heparin-like glycosaminoglycans that are isolated from animal tissues or any polysaccharides that mimic the biological activities of heparin [100]. The main property of heparin, determining its use as an anticoagulant in cardiac surgery, cardiovascular disease, cancer, autoimmune diseases, neurodegenerative diseases, and sepsis-associated coagulopathy [99,101], is its ability to bind and activate antithrombin. Heparin could be an alternative antithrombotic and anti-inflammatory median to prevent PE, especially associated to thrombophilia [78]; however, evidence on the efficacy is conflicting. To date, there is no definite evidence that antithrombotic drugs are effective for prevention of recurrence of gestational vascular complications, especially PE [79,102,103]. LMWH may have other positive impacts on the placental vascular system [103]. In particular, there is strong evidence that heparin and heparinoids may protect the eGC by interacting with N- and 6-O-sulfated HS domains [99], and act as heparinase inhibitors [104]. Another aspect is that UFH and heparan sulfate both bind to and react to cytokines, seemingly acting as messengers but potentially maintaining homeostasis by pulling the cytokines out of circulation, and reduce inflammatory background [105]. However, the side effects described for heparin and heparin-based drugs limit their use in obstetric practice. Apparently, heparin therapy complications are associated with competitive release of HS from glycocalyx by UFH; this makes endothelial cells more susceptible to ischemia and inflammatory attack during the disease progression. It is assumed that UFH has toxicity potential that we to date do not fully comprehend [105].

The majority of clinical data available on pharmacological stabilization of glycocalyx has been obtained in studies that used sulodexide, a pharmacological agent composed of a mixture of two GAGs (80% of fast-moving heparin fraction and 20% of dermatan sulfate) [106,107]. Sulodexide is characterized by a remarkable ability to be absorbed to the vascular endothelium, where it exerts an anti-thrombotic activity, restores the glycocalyx and endothelial cell permeability, modulates inflammatory and proteolytic processes, and regulates blood cell interactions with the endothelium [108]. Sulodexide promotes arterial relaxation via a mechanism involving endothelium-dependent NO production; an effect that could enhance vasodilation and decrease vasoconstriction in vascular disorders [80]. Sulodexide treatment of patients with type 2 diabetes mellitus led to an increase in systemic glycocalyx thickness and a trend towards normalization of systemic albumin clearance [107]. According to a number of authors, this drug promotes eGC reconstitution, controls eGC degrading enzymes, and poses anti-inflammatory, anti-apoptotic, and antisenescence effects on ECs [16]. However, these positive effects did not find confirmation in other studies [107]. According to a systematic review which assessed the efficacy and safety of sulodexide in chronic venous disease treatment the overall risk of adverse events was low [109].

There are few studies of the use of sulodexide in pregnancy. Normalization of the total capacity of coagulation and fibrinolytic systems in women with autoimmune hyperthyroidism was noted [81], as well as reduction of complications and improvement of perinatal outcomes in women with fetal loss syndrome on the background of thrombophilia [82]. Administration of sulodexide to rats with experimental PE throughout gestation reduced manifestations of the disease: hypertension, proteinuria, and mitochondrial dysfunction in placenta [83].

Metformin might be a promising drug for protection and regeneration of eGC. Experimental studies have shown metformin-induced recovery of endothelial glycocalyx length and density and the resulting attenuation of adhesive interactions between the endothelium and cancer cells, which might be caused by metformin impact on the nanomechanical and adhesive properties of endothelial and cancer cells in chronic hyperglycemia [110]. Previously reported cardiovascular benefits of metformin may also improve the endothelial glycocalyx. Treatment with metformin or sulodexide partly restored glycocalyx and preserved coronary microvascular function in pre-diabetic animals in a rat model of diet-induced obesity [111]. Additionally, metformin treatment was associated with an improvement of hydration of heart and kidney, and with better glycocalyx barrier properties in mice [112]. Functional in vitro and ex vivo experiments using human placental tissues demonstrated that metformin reduced endothelial dysfunction, decreased soluble fms-like tyrosine kinase 1 and soluble endoglin secretion, enhanced vasodilation in omental arteries, and induced placental angiogenesis [113].

According to a number of studies metformin has a potential to prevent or treat PE. A systematic review and meta-analysis by Kalafat E et al. (2018) provides evidence that there is a high probability that metformin use is associated with a reduced incidence of hypertensive disorders of pregnancy (HDP) especially in women with gestational diabetes and obesity, when compared with other treatments or placebo [85]. Another systematic review by Nascimento IBD et al. (2018) showed that metformfin provided greater preventive effects for pregnancy-induced hypertension, i.e., for milder hypertensive syndromes, and was less effective in PE [86]. Unfortunately, the value of mentioned reviews is limited by the small number of studies included in the analysis, the low quality of evidence, and the clinical heterogeneity precluding generalization of these results to broader populations. Nevertheless, metformin seems to be a promising drug for future application in PE, since its protective and restoring effects on eGC was well demonstrated.

The administration of glucocorticoids (hydrocortisone and methylprednisolone) has been shown to protect eGC by prevention of the endothelial perturbation and glycocalyx shedding [28,87]. Hydrocortisone stabilized mast cells, inhibited leukocyte activation, and downregulated inflammatory cytokines in experimental studies [67]. Dexamethasone suppressed the expression of MMPs and thus rescued the expression of tight junction protein 1 (ZO-1) and sdc1 in aortic homogenates of septic rats [114]. Postischemic shedding of sdc1, HS, and HA was inhibited by hydrocortisone in isolated guinea pig hearts after ischemia-reperfusion injury. Electron microscopy revealed mostly intact glycocalyx after hydrocortisone treatment [115]. Furthermore, glucocorticoids are known to promote a decrease in paracellular permeability for macromolecules.

Glucocorticoids may be used during pregnancy if the benefits outweigh the potential risk to the fetus, since these drugs easily cross placenta; therefore, it is necessary to control the symptoms of all medical conditions during pregnancy. Assessment of the glucocorticoids efficacy and safety did not find enough evidence to confirm an increased risk of pregnancy complications [116]. At the same time, any positive effect on maternal and perinatal outcomes in PE has not been noted yet, probably because glucocorticoids are not indicated for clinical use in PE. At the same time their protective effect on eGC proved in vitro may suggest their potential advantages for PE treatment.

The protective effect for eGC was demonstrated for a number of drugs and biologically active molecules in experimental studies; for some, efficacy in PE was shown. In particular, in hyperglycemia N-acetylcysteine decreased the release of HA into the bloodstream after induced hyperglycemia in healthy volunteers [117]. In an experimental study in the reduced uterine perfusion pressure model for PE in Sprague-Dawley rats, N-acetylcysteine reduced blood pressure without adversely effecting fetal weight [88]. However, according to a number of studies [89,90] and meta-analysis of randomized controlled trials [118] antioxidant therapy with N-acetylcysteine ameliorated the severity of oxidative stress in pre-eclampsia but had no effects for prevention of PE. Administration of dexmedetomidine, a sedative affecting the α2 adrenaline receptor, which is commonly used in intensive care, has been shown to improve survival and to preserve eGC in a rat heatstroke model [119]. There is some evidence that administration of dexmedetomidine in pre-eclamptic patients was associated with hemodynamic and hormonal stability, without causing severe adverse neonatal outcomes [120,121,122,123].

A potential ability to protect or restore the eGC was noted in several chemical substances: protein C, antithrombin, exogenous eGC substituents (HA and chondroitin sulfate), pentosan polysulfate, wheat germ agglutinin, rhamnan sulfate, micro RNAs, anti-TNFα agents, and synthetic antimicrobial peptides [12,16,87,99,104]. However, all these substances have been assessed only in isolated organs or animal studies; therefore, their targeted use for protection of eGC in humans cannot be recommended yet, and further research is needed [87].

Aspirin (acetylsalicylic acid, ASA) is well known to be effective for prevention of PE, and is recommended by professional societies for this purpose [2,124,125,126]. At the same time there is no available data on the direct effect of ASA on eGC. ASA, a non-steroid anti-inflammatory drug, is a strong inhibitor of cyclooxygenases (COXs). This drug’s affinity for COX-1 of platelets and for the COX-2 isoforms of endothelial cells is 95% and 5%, respectively [127]. Low doses of ASA were shown to cause anti-thrombotic effects, suggesting that inhibition of the platelets COX-1 is a specific target of ASA [128,129]. It was found that prophylactic effect of ASA is provided by its impact not only on prostaglandin biosynthesis, but especially on prostacyclin/thromboxane ratio, which is imbalanced in women with PE before the clinical onset of the disease. In normal pregnancy a vasodilator prostacyclin, produced in the endothelial cells, prevails in this ratio [130], while in PE a vasoconstrictor and platelet aggregator thromboxane outweighs. Low-dose ASA irreversibly inhibits COX-1 and, as a consequence, production of thromboxane is reliably suppressed. Only higher doses of ASA are effective to inhibit COX-2 in the blood vessel wall, and, therefore, production of prostacyclin does not respond to low-dose medication [131]. It is still not clear how these mechanisms may affect eGC.

Since one of the main factors of PE pathogenesis associated with endothelial dysfunction is decreased endothelial nitric oxide synthase/nitric oxide (eNOS/NO) activity, the effect of ASA on NO production is worth consideration. ASA, even in low dose, has shown beneficial effects mediated through NO formation, especially, through increase in haem oxygenase 1 (HO-1) and decrease in ADMA, expression in chronic stable coronary disease [132]. Therefore, the prophylactic effect of low-dose ASA in PE may originate from higher expression and enzymatic activity of HO-1 in endothelial cells, initiating the formation of carbon monoxide which acts as a vasodilator in a setting of decreased NO synthesis in PE [132,133,134]. Recent studies in experimental cell models demonstrated that ASA prevented TNF-α-mediated endothelial cell dysfunction associated with impaired vasorelaxation, angiogenesis, and trophoblast invasion, and the preventive effects were blocked by miR-155 mimic or an eNOS inhibitor. It was assumed that ASA rescued TNF-α-mediated eNOS downregulation coupled with endothelial dysfunction by inhibiting NF-κB-dependent transcriptional miR-155 biogenesis, which is involved in negative regulation of eNOS expression [135].

It looks like only one study evaluating the indirect effect of ASA on eCG, has been reported so far: the effects of platelet microparticles (PMPs) on aortic vascular endothelial injury were investigated in vitro and in vivo in experimental diabetes models. It was found that in diabetic rat model and in HUVECs, PMPs inhibited endothelial nitric oxide levels to about 50% and caused approximately twofold increase in reactive oxygen species production. Additionally, PMPs significantly decreased the eGC area and expression levels of glypican-1, and increased endothelial permeability; these effects were alleviated in the ASA treatment. It can be assumed that activated PMPs in diabetes contribute to early endothelial injury primarily due to the activation of the mTORC1 pathway, and ASA inhibits his process [136]. Impact of ASA on eGC seems to be an important subject of future research, since there is some encouraging evidence of ASA influence on the metabolism of proteoglycans and HA in normal and osteoarthritic human articular cartilage [137]. Previously reported inhibitory effect of ASA on IL-18-induced activation of cardiac fibroblasts [138] also may inspire investigation of the ASA impact on eGC, since the glycocalyx-uncovered endothelium can express and secrete more IL-18, and can enhance the devastating circle of an inflammatory reaction [139].

**Figure 1:**
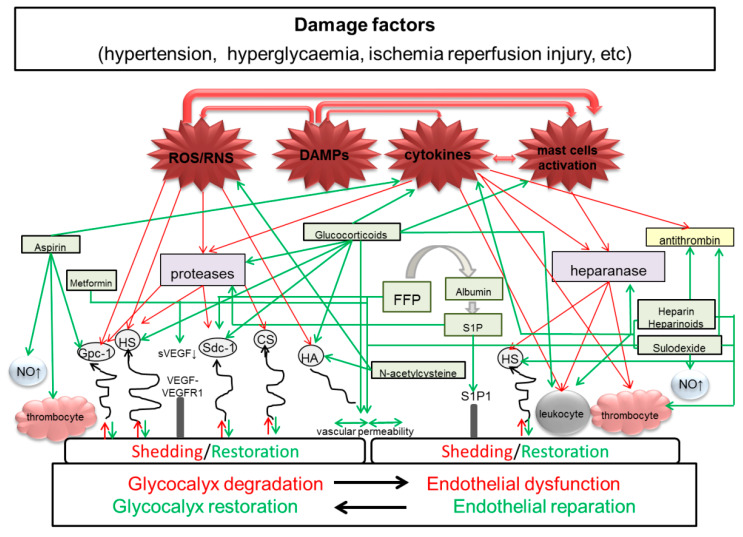
Schematic representation of possible mechanisms of the glycocalyx protection by some drugs. Damaging factors cause destruction in eGC, both directly and through various enzymes and mediators, thus impairing endothelial function, decreasing NO bioavailability, enhancing leukocyte adhesion, and increasing thrombogenic potential and endothelial permeability. Red arrows indicate activating and damaging factors and processes. Green arrows point to blocking and protective effects of drugs.

It should be noted that therapeutic strategies aimed to protect eGC are poorly understood and studied. Possible mechanisms of the glycocalyx protection by some drugs represented in Figure 1. Importance of this issue is supported by recent publications [99,140], including encompassed data on glycocalyx and its components in PE [141,142,143,144]. There is some evidence of the beneficial impact of some agents on eGC in certain conditions, and on the PE course. However, the relationship between these two benefits, as well as the impact of eGC treatment on perinatal outcomes and women’s future health, have not been assessed yet. Despite some ability to decrease eGC injury, none of these agents have been adopted as a specific eGC protector in routine clinical management of PE. Aspirin, as the most potent drug for prevention of PE, deserves special attention, particularly because ASA is known to cause a positive effect on NO production by endothelial cells and on endothelial permeability, possibly through its impact on eGC, since both NO production and permeability are eGC dependent; investigation of these associations may bring a new tool for PE prevention. Obviously, pathogenetic therapy aimed at eGC protection and restoration should be multicomponent and based on the clinical manifestation of PE and underlying or concomitant disease in mothers. Advisability of such treatment for pregnancy prolongation and reduction of short and long term complications in future must be addressed in further research.

## 2. Conclusions

Therefore, the development of organic and systemic dysfunction in moderate PE, and multiorganic/polysystemic (sometimes fatal) failure in severe PE, cannot be explained, in our opinion, without considering the damage to the eGC, which is most likely the main morphological substrate of PE. There is a number of major issues to be clarified: is SIR and its severity the key factor in the development of eGC damage and related complications? Or does the development of systemic inflammation superimpose the existing glycopathology, associated with inability of adequate eGC regeneration? Do the existing indirect methods really reflect the destructive processes in eGC in vivo? Since there are other mechanisms of endogenous NO synthesis stimulation, is disruption of shear-induced NO production due to damage and destruction of eCG a critical factor of hypertention development in PE? Is pathogenetic therapy aimed at eGC protection and restoration possible, and which drugs could be recommended for this? Further study of eGC in PE may provide a new insight in the pathogenesis of the disease and help address these questions.

## Figures and Tables

**Table 2:** Clinical and experiment research of glycocalyx and/or its components in pre-eclampsia.

№	Study Subject	Methods	Measurement	Findings
1	Sweden, 42 patients (11 with PE and eclampsia)	Case–control study ELISA	Plasma HA levels	Plasma HA level is increased in severe PE and eclampsia [46].
2	South Africa, 84 patients (28 with PE)	Dimethyl-methylene blue assay	Urinary HSPGs and CSPGs levels	Urinary excretion of HSPGs was significantly increased in the PE group compared to the normotensive pregnant group and the hypertensive nonproteinuric group [44]
3	Italy, 118 patients (93 with hypertensive disorders in pregnancy, 32 with PE)	Case–control study Lectin histochemistry	Intensity of staining of carbon residues in the glycans with lectins in the placental tissue	Various alterations of the carbohydrate metabolism and GC compositions in the placentas from women with hypertensive disorders indicate correlation with the placental morpho-functional changes, characteristic for these complications, and with the degree of clinical severity [47].
4	Germany, 55 patients (17 with HELLP-syndrome)	Observational study ELISA	GC components (sdc1, HS, and HA) were measured in serum	Increased serum levels of HS and HA were only detected in patients with HELLP. Considerable amounts of sdc1 are released into maternal blood during uncomplicated pregnancy. The HELLP syndrome is associated with an even more pronounced shedding of GC components. Maternal vasculature as well as placenta may be a possible origin of circulating GC components [48].
5	Germany, 16 patients (8 with HELLP-syndrome)	Case–control study Immunohistochemistry Electron microscopy	Visualization and expression assessment of GC components (sdc1, HS, HA) in placenta	Large amounts of sdc1 were found, but neither HA nor HS as the major components. Intravillous fetal endothelium did not express any of the investigated GAGs. Healthy women and patients with HELLP showed no differences concerning GC composition and thickness of the syncytiotrophoblast [49].
6	UK, 75 patients (17 with PE)	ELISA Glycosaminoglycan assay Immunohistochemistry	Concentration and expression sdc1 and sulfated GSGs in placental tissues	Decreased sGAGs and sdc1 in PE were not related to labor, gestational age, and birthweight centile [50].
7	Brazil, 153 patients (60 with PE)	ELISA	Serum HA levels	Increased release of HA may contribute to an elevated pro-inflammatory response and tissue damage in women with PE [51].
8	Turkey, 81 patients (49 with PE)	A cross-sectional study ELISA	Serum endocan levels	Mean endocan levels were not significantly different among groups [42].
9	China, 22 patients (12 with PE)	Case–control study Immunohistochemistry; qRT-PCR; Western blotting; ELISA	Immunohistochemistry was used to evaluate the location of endocan. Then, the mRNA and protein levels of endocan in placenta were detected using qRT-PCR and Western blotting. Serum endocan concentration was measured by ELISA	Expression of endocan mRNA and protein were increased in the placenta tissues of PE compared with in the normal pregnancy; however, the endocan concentration of maternal serum did not differ significantly [43,52].
10	Brazil, 117 patients (50 with PE)	Observational and case–control study MagPlex(TH)-C	Plasma endocan-1 levels	Endocan-1 is increased in women with PE. The negative correlations between endocan-1 and clinical data suggest that this molecule may also be involved in prematurity and low birth weight [53].
11	USA, 506 patients (130 with uncomplicated pregnancy; 102 with PE; 274 with other great obstetrical syndromes)	A cross-sectional study ELISA	Plasma endocan-1 concentrations	Median maternal plasma endocan concentrations were higher in PE patients and lower in acute pyelonephritis with bacteremia than in uncomplicated pregnancy. No significant difference was observed in the median plasma endocan concentration between other great obstetrical syndromes and uncomplicated pregnancies. The difference in changes of endocan in PE and acute pyelonephritis with bacteremia may confirm that the two diseases differ in pathogenetic mechanisms, despite their associations with systemic vascular inflammation and endothelial cell activation/dysfunction [54].
12	Russia, 23 patients (16 with moderate and severe PE)	Case–control study Lectin histochemistry	The study of carbohydrate phenotype of placenta was carried out by the lectin staining of syncytiotrophoblast membranes and the membranes of endothelial cells of terminal placental villi	The most prominent alteration of the GC composition was found in the placentas of women with severe PE. The modified glycome of syncytiotrophoblast and capillary endothelium may play an important role in pathogenesis of PE [55].
13	Canada, 28 patients (14 with PE)	Retrospective and case–control study qRT- PCR; situ hybridization; ELISA	Decorin expression was measured at tissue and cell levels in the placenta sections. Retrospective measurements of plasma decorin levels during the second trimester were carried out.	Decorin overexpression by basal decidual cells is associated with hypoinvasive phenotype and poor endovascular differentiation of trophoblast cells in PE. Elevated plasma decorin concentration is a potential predictive biomarker for PE before the onset of clinical signs [56].
14	Turkey, 129 patients (99 with PE)	A cross-sectional study ELISA	Serum endocan-1 concentrations	Serum endocan concentrations were significantly elevated in women with PE versus normotensive controls, and concentrations seemed to be associated with the severity of the disease [57].
15	Serbia, 44 patients (14 with PE + IUGR)	Case–control study DSA-FACE method; electrophoresis; lectin and immunoblotting; lectin affinity chromatography	N-glycan analysis in placenta	Glycans on placental membranes were altered due to PE [58].
16	USA, longitudinal study (*n* = 8); cross-sectional 3rd trimester study (34 patients, 17 with PE); case–control study (44 patients (19 with PE)	Case–control, longitudinal, and cross- sectional studies. ELISA Isolation and analysis of placental RNA Placental immunohistochemical staining and scoring	Plasma sdc1 levels and placental sdc1 expression	Soluble sdc is significantly lower before the clinical onset of PE, with reduced expression of sdc1 in the placenta after expulsion, suggesting a role of GC disturbance in PE pathophysiology [59].
17	Turkey, 80 patients (27 with EO- PE and 27 LO- PE)	Cross-sectional study ELISA	Serum sdc1 levels	Control group presented significantly higher sdc1 levels, than EO and LO-PE [52].
18	Brasil, 60 patients (20 with PE)	ELISA	Plasma HA levels	Significantly higher plasma levels of HA in PE than in normotensive pregnant women and non-pregnant women, suggesting involvement of HA as DAMPs in SIR [60].
19	USA, 137 women (14 with EO-PE, 29 with LO-PE)	ELISA and noninvasive sublingual eGC measurements by sidestream dark field imaging	Plasma levels of sdc1, HA, HSPGs, perfused boundary region (width of the eGC that was permeable to RBCsreflects eGC degradation) and the percentage of vessels that were filled with RBCs ≥50% of the time (this reflects a microvascular perfusion)	In LO-PE the structural eGC changes (eGC degradation, larger perfused boundary region) was higher and percentage of vessels that were filled with RBCs was significantly lower) were accompanied by elevated plasma concentration of eGC components [61].
20	Turkey, 78 women (25 with EO-PE and 16 with LO-PE)	ELISA	Plasma endocan levels	There was no significant difference between endocan levels in EO-PE or LO-PE compared with their corresponding control groups, nor between EO- and LO-PE groups [62].
21	Poland, 60 women (20 with EO-PE and 20 with LO-PE)	ELISA	Serum HA and sdc1 levels	Concentration of HA was significantly higher and the level of sdc1 was significantly lower in patients with EO and LO-PE than in the control group [63].
22	Austria, single center nested case–control study, 107 patients (95 with normal pregnancy, 12 with PE)	ELISA	Serum sdc1 levels were measured at 10 dynamic points during pregnancy	Sdc1 levels were lower in women developing PE compared to normal pregnancies, and sdc-1 might be useful to predict PE. After delivery, sdc1 levels remained higher in women with PE [64].

**Table 3:** Pharmacological protection and regeneration of the endothelial glycocalyx (eGC).

Drugs/Molecules	Effects	Application in Pre-Eclampsia
Fresh frozen plasma	Improves junctional integrity of endothelial cells, partially restores eGC and preserves endothelial sdc1 [69,70]	Limited data on application in severe pre-eclampsia-eclampsia with HELLP syndrome [71,72]
Albumin	Reduces eGC shedding and edema formation and improves endothelial integrity [73]	No impact on blood pressure and renal function, uteroplacental, and fetoplacental resistance [74]. Used for fluid resuscitation in PE, prior to regional anesthesia for caesarean section, compensation of hemorrhagic blood loss in labor [75]. Caused positive effect in a patient with severe PE complicated with postpartum massive ascites and pleural effusion [76]
Heparin	Maintains the eGC thickness, inhibits neutrophil adherence and inflammation [77]	Is effective in PE and concomitant inherited or acquired thrombophilias [78]. According to meta-analysis, LMWH does not seem to reduce the risk of recurrent placenta-mediated pregnancy complications in at-risk women [79].
Sulodexide	Increases GAGs synthesis [16] and promotes arterial relaxation [80]	There are few reports on effective use during pregnancy [81,82]. Decrease in the PE symptoms (lower blood pressure and less proteinuria) in experimental PE in rats [83].
Hydrocortisone Methylprednisolone	Protect eGC by prevention of endothelial perturbation and glycocalyx shedding [28]	No positive effect on maternal and perinatal outcomes in PE
Metformin	Improves the eGC barrier properties [84]	According to several studies, metformin may prevent or treat PE [85,86]
N-acetylcystein	Preserves eGC [87]	Beneficial effect of the N-acetylcystein administration was noted in some clinical and experimental studies with PE [88,89,90]

## Abbreviations

ADMA	asymmetric dimethylarginine
AECA	anti-endothelial cell antibodies
APS	antiphospholipid antibodies
ASA	aspirin, acetylsalicylic acid
CAMs	cell adhesion molecules
CNS	central nervous system
COX	cyclooxygenase
CSPGs	chondroitin sulfate proteoglycans
DAMPs	damage-associated molecular patterns
DIC	disseminated intravascular coagulation
DSA-FACE	DNA sequencer-assisted fluorophore assisted carbohydrate electrophoresis
eGC	endothelial glycocalyx
eNOs	endothelial nitric oxide synthase
ec-SOD	extracellular superoxide dismutase
ELISA	enzyme-linked immunosorbent assay
EO-PE	early-onset pre-eclampsia
FFP	fresh frozen plasma
GAGs	glycosaminoglycans
GC	glycocalyx
GMB	glomerular basement membrane
HA	hyaruronan, hyaluronic acid
HELLP	Hemolysis, Elevated Liver enzymes and Low Platelet count
HO-1	haem oxygenase 1
HS	heparan sulfate
HSPGs	heparan sulfate proteoglycans
HUVECs	human umbilical vein endothelial cells
LMWH	low-molecular-weight heparin
LO-PE	late-onset pre-eclampsia
MMPs	matrix metalloproteinases
NO	nitric oxide
PE	pre-eclampsia
PMPs	platelet microparticles
qRT-PCR	quantitative real-time polymerase chain reaction
RBCs	red blood cells
SIR	systemic inflammatory response
S1P	sphingosine-1-phosphate
S1P1	sphingosine-1-phosphate receptor 1
sdc1	syndecan 1
UFH	unfractionated heparin
